# Fatal Asphyxia Potentially Caused by COVID-19-Induced Exacerbation of Pre-existing Tracheal Stenosis

**DOI:** 10.7759/cureus.34246

**Published:** 2023-01-26

**Authors:** Masaatsu Kuwahara, Hiroko Otagaki, Hideaki Imanaka

**Affiliations:** 1 Department of Emergency Medicine, Takarazuka City Hospital, Takarazuka, JPN

**Keywords:** cricothyrotomy, tracheal stenosis, intubation, covid-19, asphyxia

## Abstract

We report a case of cardiac arrest due to asphyxia caused by coronavirus disease 2019 (COVID-19) in a patient with no history of tracheal intubation but with a history of subglottic stenosis. A 54-year-old man suffered a cardiac arrest at home. The patient had tracheal stenosis; therefore, it was difficult to intubate. The patient had COVID-19, which was presumed to have aggravated the existing tracheal stenosis and caused asphyxiation. The patient died seven days later. This is, to our knowledge, the first report of a patient with subglottic stenosis potentially aggravated by COVID-19, resulting in asphyxia-related cardiopulmonary arrest. The patient could not be saved, but emergency physicians should be aware that airway obstruction can be caused by viral infections, including severe acute respiratory syndrome coronavirus 2 infections. Physicians should consider the difficulty in performing oral intubation and cricothyrotomy and be aware of alternative methods to secure the airway.

## Introduction

A novel coronavirus was identified as the cause of a pneumonia outbreak in Wuhan, Hubei Province, China at the end of 2019. It spread rapidly, first gaining endemic status in China and, subsequently, a pandemic status. In February 2020, the World Health Organization named the disease coronavirus disease 2019 (COVID-19). The virus causing COVID-19 is the severe acute respiratory syndrome coronavirus 2 (SARS-CoV-2).

Tracheal stenosis is the main side effect among patients with COVID-19 undergoing tracheal intubation [1]. To our knowledge, only one case of tracheal stenosis has been reported in a patient with COVID-19 who did not undergo tracheal intubation [2]. We report a case of cardiac arrest due to asphyxia caused by COVID-19 in a patient with no history of tracheal intubation but with a history of subglottic stenosis.

## Case presentation

**Figure 1:**
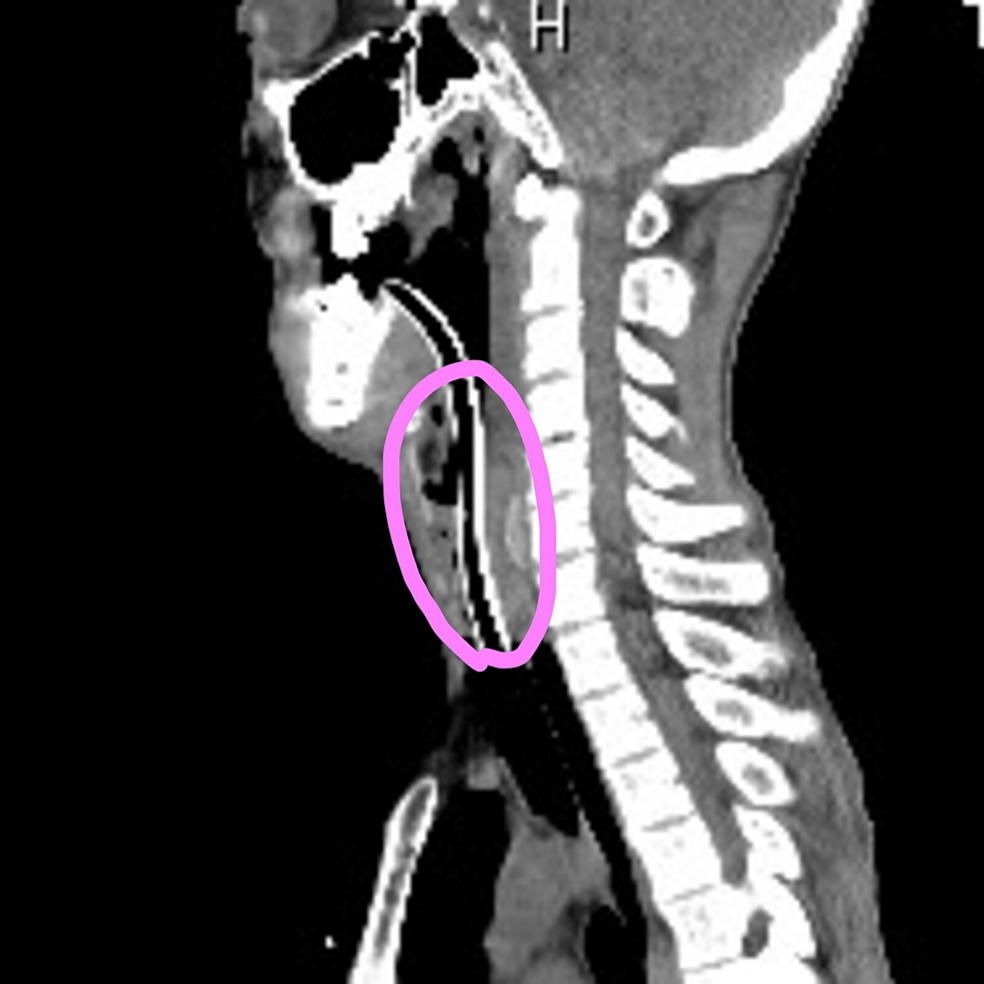
Cervical CT, lateral view. Post-return of spontaneous circulation CT reveals marked stenosis (50 mm in length) of the trachea below the glottis.

A 54-year-old Asian man collapsed at home after complaining of breathing difficulty. A family member witnessed the incident, and an ambulance was immediately called. Resuscitation was immediately initiated by the emergency medical services team, and, as soon as a route was secured, the patient was transported to the hospital while receiving chest compressions and adrenaline administration. The asystole electrocardiogram waveform was confirmed upon arrival at the hospital. As the patient did not respond to bag-valve-mask ventilation, our treatment strategy was to intubate the trachea. First, we attempted oral intubation. However, neither an 8- nor a 7-mm tube could be inserted through the glottis due to strong resistance. Therefore, we performed a cricothyrotomy. However, the trachea was markedly narrowed just below the cricothyroid ligament, prohibiting tracheal tube insertion. We reverted to oral intubation, inserting a 6-mm uncuffed tube. Despite strong resistance, we successfully intubated the patient, yielding a return of spontaneous circulation (ROSC). Post-ROSC CT revealed marked stenosis (50 mm in length) of the trachea below the glottis (Figure 1).

Despite the small diameter and uncuffed nature of the tracheal tube, no cuff leakage was observed during ventilation, and the patient was judged to have severe stenosis, with his trachea in close contact with the tube.

**Figure 2:**
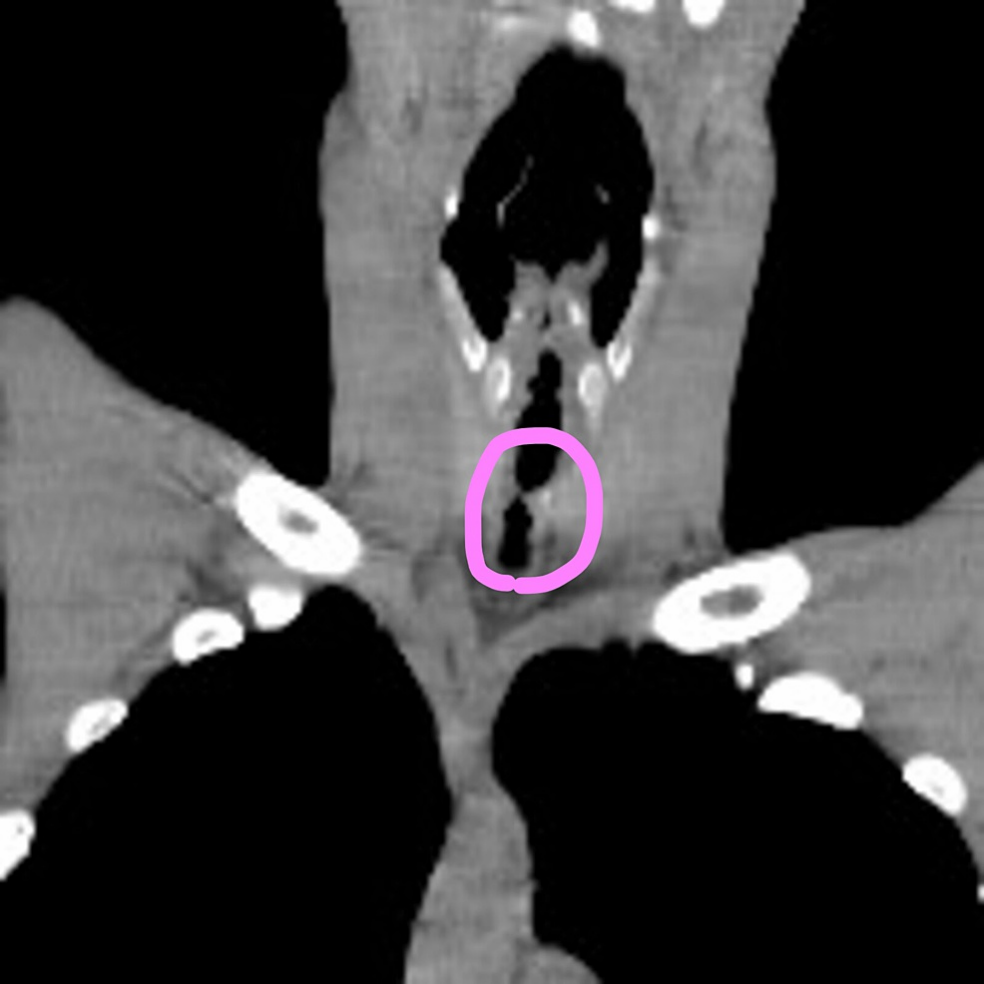
Cervical CT, lateral view. The patient’s medical records revealed that he had been diagnosed with benign subglottic stenosis three years earlier. At the time, the lumen was narrowed to 9 mm over a length of 46 mm, from the subglottis to the caudal side of the trachea.

The patient’s medical records revealed that he had been diagnosed with benign subglottic stenosis three years earlier. At that time, the lumen was narrowed to 9 mm over a length of 46 mm, from the subglottis to the caudal side of the trachea (Figure 2).

He had been advised to regularly visit the hospital for check-ups, but he did not do so. Therefore, the extent of his tracheal stenosis just before arriving at our hospital is unclear.

He had been experiencing worsening respiratory distress symptoms for several days. Antigen quantification testing upon arrival at the hospital revealed that the patient had a COVID-19 infection. Based on the course of events, we believe that his subglottic stenosis was aggravated by COVID-19, causing asphyxia, which, in turn, led to cardiopulmonary arrest.

The patient was treated in the intensive care unit, including temperature management therapy, for post-resuscitation syndrome, but died on the seventh day of hospitalization.

## Discussion

Subglottic stenosis is an obstruction of the central airway in the region from the glottis to the tracheal ring [3]. Several etiologies have been proposed for subglottic stenosis, with the most common being prolonged intubation, excessive endotracheal tube cuff pressure, and tracheostomy-related trauma. Other causes, including infections, gastroesophageal reflux disease, systemic diseases, radiation therapy, inhalation injury, occupational exposure, inflammatory bowel disease, primary and secondary tracheal malignancies, and congenital diseases, are less common [4]. In recent years, autoimmune diseases have also been linked to this condition [5]. Viral upper respiratory tract infections are believed to play an important role in causing acute exacerbations of airway obstruction [6]. Pathological results suggest that SARS-CoV-2 may be directly responsible for tracheal stenosis [7].

The extent of the patient’s subglottic stenosis immediately prior to the onset of COVID-19 is unknown because he had not gone for a check-up at that time. However, the SARS-CoV-2 infection was likely a contributing factor in causing acute exacerbation of the condition. Nevertheless, further research is needed to investigate this conjecture. To our knowledge, this is the first report of a case of subglottic stenosis likely aggravated by COVID-19 and subsequently causing cardiac arrest.

In the present case, a normal-sized tube could not be used for tracheal intubation because of the patient’s subglottic stenosis. Therefore, a cricothyrotomy was performed, but, even at that level, the lumen of the trachea was markedly narrowed, preventing the insertion of a tracheostomy tube. In a previous case in which cricothyrotomy was unsuccessful because of the location of subglottic stenosis, retrograde intubation was successfully performed [8]. In this case, we inserted a pediatric (6 mm diameter), cuffless tube. If this procedure also fails, the remaining options are retrograde intubation, intubation with a fiberscope, or tracheotomy with a percutaneous tracheostomy kit. Emergency physicians should be aware that securing an airway via cricothyrotomy may be impossible in patients with subglottic stenosis, in which case the above methods should be considered.

## Conclusions

This is, to our knowledge, the first report of a patient with subglottic stenosis potentially aggravated by COVID-19 and resulting in asphyxia-related cardiopulmonary arrest. Unfortunately, we could not save the patient in this case, but emergency physicians should be aware that airway obstruction can be caused by viral infections, including SARS-CoV-2 infection. In such cases, physicians should keep in mind that oral intubation and cricothyrotomy may be difficult to perform and be aware of alternative methods to secure the airway.
